# Benchmarking Surgeons’ Gender and Year of Medical School Graduation Associated With Monthly Operative Workdays for Multispecialty Groups

**DOI:** 10.7759/cureus.25054

**Published:** 2022-05-16

**Authors:** Franklin Dexter, Richard H Epstein, Johannes Ledolter, Amy C Pearson, Joni Maga, Brenda G Fahy

**Affiliations:** 1 Anesthesia, University of Iowa, Iowa City, USA; 2 Anesthesiology, University of Miami Miller School of Medicine, Miami, USA; 3 Business Analytics, University of Iowa, Iowa City, USA; 4 Anesthesiology, Advocate Aurora Health, Illinois, USA; 5 Perioperative Medicine and Pain Management, University of Miami Miller School of Medicine, Miami, USA; 6 Anesthesiology, University of Florida College of Medicine, Gainesville, USA

**Keywords:** surgery, operating room management, medical group administration, managerial epidemiology, gender bias

## Abstract

Background

Female surgeons reportedly receive less surgical block time and fewer procedural referrals than male surgeons. In this study, we compared operative days between female and male surgeons throughout Florida. Our objective was to facilitate benchmarking by multispecialty groups, both the endpoint to use for statistically reliable results and expected differences.

Methodology

The historical cohort study included all 4,060,070 ambulatory procedural encounters and inpatient elective surgical states performed between January 2017 and December 2019 by 8,472 surgeons at 609 facilities. Surgeons’ gender, year of medical school graduation, and surgical specialty were obtained from their National Provider Identifiers.

Results

Female surgeons operated an average of 1.0 fewer days per month than matched male surgeons (99% confidence interval 0.8 to 1.2 fewer days, P < 0.0001). The mean differences were 0.8 to 1.4 fewer days per month among each of the five quintiles of years of graduation from medical school (all P ≤ 0.0050). Results were comparable when repeated using the number of monthly cases the surgeons performed.

Conclusions

An average difference of ≤1.4 days per month is a conservative estimate for the current status quo of the workload difference in Florida. Suppose that a group’s female surgeons average more than two fewer operative days per month than the group’s male surgeons of the same specialty. Such a large average difference would call for investigation of what might reflect systematic bias. While such a difference may reflect good flexibility of the organization, it may show a lack of responsiveness (e.g., fewer referrals of procedural patients to female surgeons or bias when apportioning allocated operating room time).

## Introduction

Fundamentally, growing surgical suites and caring for more surgical patients depend on maintaining or increasing surgeons’ caseloads, which, in turn, depends on hospitals supporting these surgeons’ productivity. In many large multispecialty groups, such support is achieved through a combination of incentive-based compensation (e.g., calculated using United States’ relative value units) and convenient access to the operating room schedule. However, according to federal anti-discrimination statutes, organizations must ensure that neither incentive programs nor surgical scheduling, directly or indirectly, are biased with respect to the protected characteristics of the employees, including gender or age [1]. Such assurance is achieved, at least in part, through the use of compliance policies and formal vetting, as well as through the review of managerial decision-making to obviate bias. Another process used by management (e.g., surgeons and anesthesiologists in leadership roles) to assess the presence of potential bias related to surgical activity is to compare surgeons’ numbers of operating room days, their access to the first case of the day times, and other elements related to productivity.

In December 2020, Yesantharao et al. reported that at three affiliated teaching hospitals in the United States, female surgeons were less likely to have adequate block time than their male colleagues in terms of hourly workloads [2]. (Genders listed in reference [2] are binary: “female” and “male.” Throughout our paper, we use “female” and “male” because those are the terms in the cited papers and are the only options in the data.) In November 2021, Dossa et al. reported that throughout Ontario, female surgeons received fewer procedural referrals than male surgeons, principally because male physicians more often referred patients to male surgeons rather than female surgeons [3]. In this study, we used surgical cases statewide in Florida, categorized by surgeon and date of surgery, to compare operative days between female and male surgeons. Our managerial epidemiology study adds baseline information regarding the possible gender-related differences in surgical activity, allowing large group practices (such as those of the authors, Universities of Iowa, Florida, and Miami) to evaluate whether they are an outlier compared to other organizations. The goal of our study was not to explain the potential reasons for gender disparity, as assessment and change would be made within organizations. Rather, our objective was to facilitate benchmarking by multispecialty groups. To compare female and male surgeons, multispecialty groups need to know what endpoint to use for statistically reliable results. In addition, multispecialty groups need to know what differences may be expected for that endpoint. We address both of these requirements.

## Materials and methods

The Institutional Review Board of the University of Florida (IRB202002442) approved this research as exempt from patient consent. The Institutional Review Boards of the University of Iowa (October 20, 2021) and the University of Miami (October 21, 2021) determined that the current analyses of deidentified data did not meet the regulatory definition of human subjects research. Authors from the University of Iowa (FD, JL) performed formal analyses and corresponding statistical programming. An author from the University of Miami (RHE) performed database programming and data curation. This study follows the relevant portions of the STrengthening the Reporting of OBservational studies in Epidemiology (STROBE) checklist for cohort study [4].

State of Florida data

We used publicly available data from the Agency for Health Care Administration for patients receiving care at non-federal hospitals in Florida from January 1, 2017, through December 31, 2019. The federal hospitals are not included in the data because US federal hospitals are not regulated by the states. After special approval by the Agency for Health Care Administration, these data were supplemented with the date of each encounter (for ambulatory patients) and the date of each hospital admission (for inpatients). Dates were necessary to obtain our primary endpoint, that is, the count of days per month that each surgeon performed at least one elective case. Data use agreements were executed between the University of Florida and the Agency for Health Care Administration and between the University of Florida and the University of Miami. The Agency for Health Care Administration disclaims responsibility for the results and conclusions of this study.

**Figure 1:**
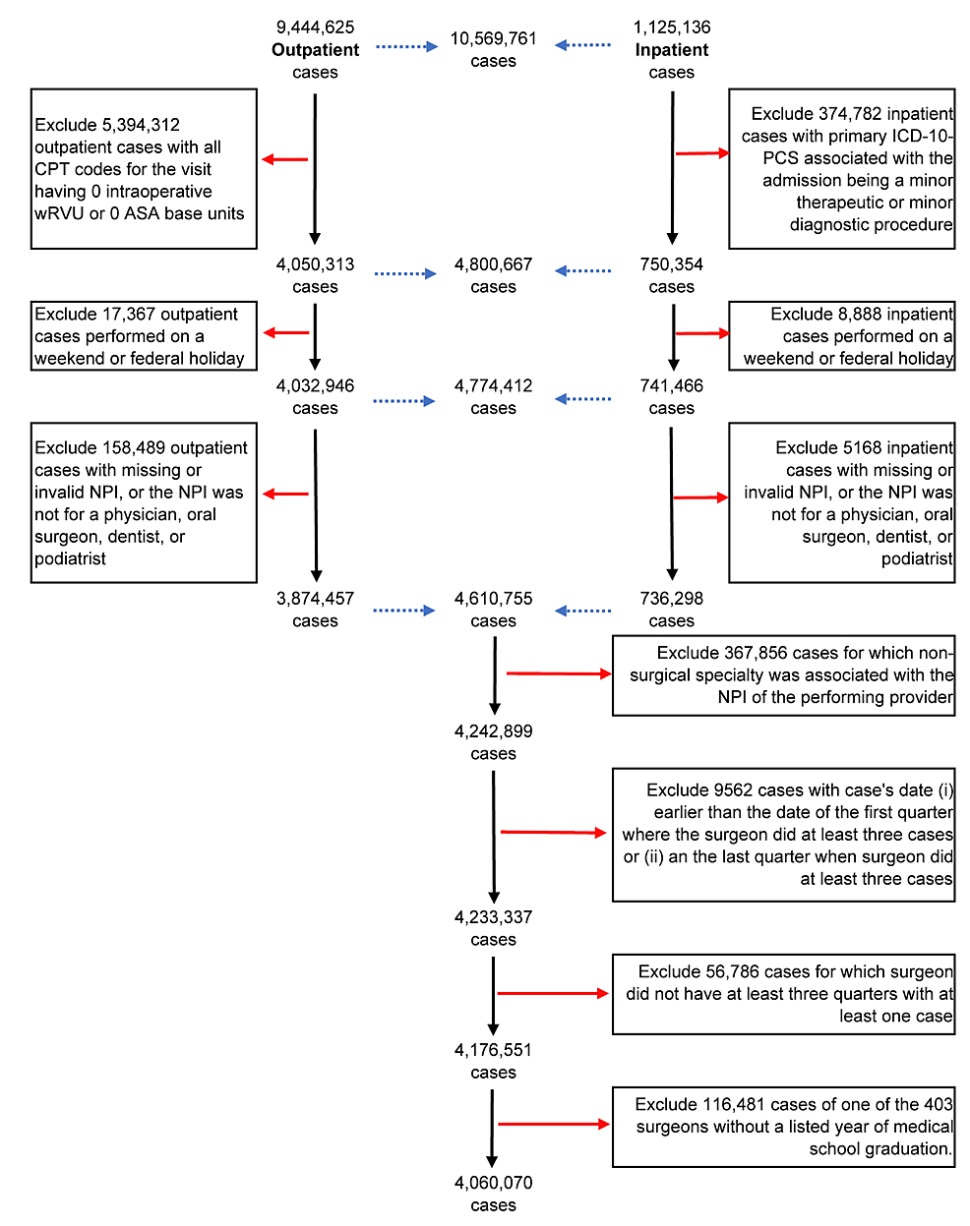
Elective case exclusions from the 287 hospitals and 440 ambulatory surgery centers in Florida, 2017-2019. ASA: American Society of Anesthesiologists; CPT: Current Procedural Terminology; ICD-10-PCS: International Classification of Diseases, Tenth Revision, Procedure Coding System; NPI: National Provider Identifier; wRVU: intraoperative work relative value units

Figure 1 lists the exclusion criteria among the 10,589,761 outpatient procedural encounters and inpatient elective surgical admissions in Florida between January 1, 2017, and December 31, 2019. There were 4,176,551 cases, performed by 8,875 surgeons, with female or male determinable from the National Provider Identifier database [5]. The National Provider Identifier demographic information is submitted by the provider or the provider’s institution, and gender was collected as male or female. We excluded 403 surgeons without a listed year of medical school graduation (i.e., from a school of allopathic, osteopathic, dental, or podiatric medicine), accounting for 116,481 cases. The final population studied was 4,060,070 elective surgical cases performed at 609 facilities by 8,472 surgeons, 20.4% (1,724) of whom were female. The surgeon’s self-reported principal specialty was used for classification (e.g., colorectal and oral maxillofacial surgery). Tables listing counts of surgeon-quarters are in Stata output at https://FDshort.com/SurgFMYr. The state data are by quarter, but analyses are reported per month.

Statistical analyses

The full linear regression Stata outputs, including commands and statistical annotation, are available at https://FDshort.com/SurgFMYr, the file listed in the same sequence as in the Methods and Results (StataCorp, College Station, TX, USA). Multispecialty groups aiming to apply our results would not need to use these methods. Instead, they can rely on the endpoint of operative days per month (see Discussion) and calculate the average difference between male and female surgeons. For those interested in the results and their application, instead of methodology, we have written the Results section to be self-contained (i.e., this section can be skipped).

For the regress command, the dependent variable was the surgeon’s number of operative days per month during the quarter. The independent variables included (a) the 10 combinations of gender and quintile of the year of graduation from medical school [6], (b) the 16 different surgical specialties studied, and (c) the 12 sequential quarters from January 2017 to December 2019 as categories (because of non-monotonic association with dependent variable). Robust variance estimation was used for potential model misspecification, calculated with clustering using the 609 facilities [7]. There were 90,182 combinations of surgeons and quarters included in the regression. We used fixed-effects modeling rather than creating a mixed-effects model because the latter would have included three non-nested random effects (surgical specialty, quarter, and facility) and the data were highly unbalanced (e.g., many more orthopedic surgeons than gynecological oncologists). Estimated coefficients of the regression model and their standard errors are reported in the Stata output at https://FDshort.com/SurgFMYr.

Contrasts of predictive margins were calculated by (a) gender and (b) gender for each of the five quintiles of graduation year using the Stata margins command. The other variables of quintile of graduation year, specialty, and quarter were handled using their average estimates over all observations. Standard errors for the contrasts were calculated using the delta method when applying the regression model’s variance estimates. The standard errors were used to produce corresponding 99% confidence intervals (CIs). These contrasts are reported in the paper because they combine the coefficients of gender alone with its interactions.

Similar to sensitivity analysis, the preceding calculations were repeated using robust regression, the bi-weight Huber tuning constant being set to the default value of the Stata command (rreg), seven times the median absolute deviation from the median residual [8]. Overall, 0.43% of the 90,182 observations had weights less than 0.10, and 0.19% had weights less than 0.01 (i.e., few exclusions). Contrasts of predictive margins were then calculated using the delta method from the robust regression estimates and standard errors.

Our sample size of surgeons was limited in being from a single state, albeit the third most populous state in the country. The last date of our sample, December 31, 2019, was the most recent available at the time of analysis. The first date, January 1, 2017, was selected to reflect contemporaneous operating room management practice. To evaluate whether our sample size of three years was adequate, we checked that the 99% CI widths for contrasts between genders were comparable to or smaller than the managerially significant difference of two workdays per month. The threshold of two workdays is important, not managerially ad hoc, because the least amount of practical surgeon block time while maintaining desired waiting times for patients is once every two weeks [9,10]. Similarly, to evaluate the importance of the effect size of estimated contrasts between genders, we made comparisons using Cohen’s d [11]. The numerator was the estimated contrast between genders. The denominator was the pooled standard deviation, pooling among surgeons of the same specialty, quarter, gender, and facility [11].

Comparisons of surgeons by gender based on our secondary endpoint of cases per month were limited by copious outliers, in contrast to our analysis by the number of operative workdays. In other words, while there are a maximum number of monthly regular workdays (i.e., 19 to 23, depending on the month, reduced by the number of holidays and clinic days needed for preoperative and postoperative evaluations), there are orders of magnitude differences among surgeons in their monthly caseloads. Sources of absences of limits on caseload include surgeons using multiple operating rooms on the same day (e.g., operating with a surgical fellow, senior resident, or other qualified first assistants), performing many brief procedures (e.g., ophthalmologists specializing in cataract surgery), or having multiple nurse practitioners and physician assistants to help care for patients pre- and postoperatively. Notwithstanding these limitations, we repeated the analyses based on cases per month and included those results as sensitivity analyses.

## Results

Adjusting for covariates such as specialty, female surgeons operated an average of 1.0 fewer days per month than male surgeons (99% confidence interval 0.8 to 1.2 fewer days, P < 0.0001; Table 1). Using the pooled standard deviation of 2.60 days per month, Cohen’s d was 0.39, showing a small-to-medium effect size difference due to gender [11]. The mean differences were 0.8 to 1.4 fewer days per month among each of the five quintiles of years of graduation from medical school (all P ≤ 0.0050; Table 1). These calculations were performed with robust variance estimation, using clustering by the facility where the surgeon worked during most of their operating room days. Repeating the analyses using robust regression, mean differences were 0.7 to 1.4 fewer days per month (all P < 0.0001) for female compared to male surgeons (Table 1). All 10 of the 99% confidence intervals were within the range of 0.5 to 1.7 fewer days per month. (That range would be for use by individual multispecialty groups when calculating differences; see Discussion.) Full sets of parameter estimates, breakdowns by specialty, years of practice, and other data are available in the Stata output at https://FDshort.com/SurgFMYr.

**Table 1:** Contrasts between male and female surgeons based on workdays per month, reported as mean difference (99% confidence interval). ^a^Includes dentists (e.g., oral maxillofacial and intraoperative dental care of pediatric patients), and thus “medical graduation” refers to the medical school or dental school year of graduation. ^b^Differences adjusted for the 16 self-reported principal specialties and the 12 sequential quarters. Parameter estimates for these combinations are available in the Stata output at https://FDshort.com/SurgFMYr, along with all other estimated coefficients of the model. The degrees of freedom for each contrast are given in the Stata output. ^c^Results using ordinary least square regression were comparable to estimates and confidence intervals of the robust regression; see the Stata output.

Contrast of male versus female surgeons, stratified by year of medical graduation^a^	Robust variance estimated with clustering by facility^b^	Robust regression^b,c^
Overall	1.0 (0.8 to 1.2), P < 0.0001	1.0 (0.9 to 1.1), P < 0.0001
1954 to 1983	0.8 (0.1 to 1.6), P = 0.0050	0.7 (0.5 to 1.0), P < 0.0001
1984 to 1991	0.9 (0.5 to 1.4), P < 0.0001	0.9 (0.7 to 1.1), P < 0.0001
1992 to 1998	1.4 (1.0 to 1.7), P < 0.0001	1.4 (1.2 to 1.5), P < 0.0001
1999 to 2005	1.0 (0.6 to 1.3), P < 0.0001	1.0 (0.9 to 1.1), P < 0.0001
2006 to 2018^a^	1.0 (0.7 to 1.2), P < 0.0001	0.9 (0.8 to 1.0), P < 0.0001

Our secondary endpoint was the number of monthly cases the surgeon performed. Results were comparable to the primary analysis (Table 2), showing the validity of the primary result. However, there was sensitivity to the method of analysis and there were larger standard errors, both as expected (see Discussion).

**Table 2:** Contrasts between male and female surgeons based on cases per month, reported as mean difference (99% confidence interval). ^a^The data include dentists (e.g., oral maxillofacial surgeons and dentists who performed intraoperative dental care for pediatric patients). ^b^The differences were adjusted for the 16 self-reported principal specialties and the 12 sequential quarters. There are multiple outliers in cases per month; therefore, the robust regression may be more reliable. However, the robust regression neglects potential correlations among surgeons from the same facility. The substantially wider confidence intervals than for Table 1 reflect the data and were expected. Treat the table solely as a sensitivity analysis for Table 1. Regardless, for details, the two linear regression models are provided with all estimated coefficients in the Stata output at https://FDshort.com/SurgFMYr.

Contrast of male versus female surgeons, stratified by year of medical school graduation^a^	Robust variance estimated with clustering by facility^b^	Robust regression^b^
Overall	4.3 (2.8 to 5.8), P < 0.0001	2.1 (1.8 to 2.3), P < 0.0001
1954 to 1983	4.3 (0.3 to 8.3), P = 0.0061	1.4 (0.9 to 2.0), P < 0.0001
1984 to 1991	4.8 (2.1 to 7.4), P < 0.0001	1.9 (1.5 to 2.2), P < 0.0001
1992 to 1998	5.0 (1.2 to 8.9), P = 0.0008	3.5 (3.2 to 3.8), P < 0.0001
1999 to 2005	5.3 (3.3 to 7.2), P < 0.0001	2.0 (1.8 to 2.3), P < 0.0001
2006 to 2018^a^	2.1 (0.7 to 3.5), P = 0.0001	1.4 (1.2 to 1.7), P < 0.0001

## Discussion

Magnitude of differences to consider during investigation

Suppose that a multispecialty group’s female surgeons are operating, on average, three fewer days per month than the group’s male surgeons, paired by specialty. Our results from Florida show that such a large average difference between male and female surgeons calls for investigation of what might reflect systematic bias. The multispecialty group with three fewer days per month for female surgeons should seek to determine whether the difference reflects good flexibility of the organization (e.g., in meeting requests of women who want to work fewer days per month for personal or professional reasons) versus lack of responsiveness by the organization (e.g., fewer referrals of procedural patients to female surgeons as in Ontario [3], bias among department heads or other responsible leadership when apportioning their service’s allocated operating room time, the bias of female surgical schedulers and clinic nurses [12], and insufficient provision of administrative staff, nurse practitioners, and physician assistants).

An important feature of our study is that the data are cross-sectional, accurately highlighting organizations’ current differences when using managerial data to screen for potential (i.e., unusually large) gender-based bias in operating room management practices and policies related to surgical productivity. Thus, our study was designed to be useful for medical directors and department or division heads responsible for organizations’ perioperative services. However, our results are not intended to be used to forecast surgeons’ scheduling patterns over future years (e.g., not for local facility capacity planning).

An average difference of 1.2 or fewer days per month should be regarded as the status quo (P < 0.0001), a difference that is substantive and classified as small to medium [11]. An organization with differences larger than 1.7 days per month may not have systematic bias, but rather differences that may simply reflect personal preferences. Surgeons of different demographic generations may have different organizational behaviors and personal life priorities. The goal of our research was to make it practical for healthcare leaders and administrators to evaluate their own organizations. The need for evaluations is being realistic, considering that at least 36% of facilities exceed the threshold, based on a 99% two-sided Clopper-Pearson confidence interval for the 32 of 56 with at least 96 surgeon quarters both for female and male surgeons.

Do not perform benchmarking by counts of cases

When preparing our Institutional Review Board application, we expected that analyses by caseload per month would be fraught with impractically large variability for individual multispecialty groups, nonetheless statewide. That was precisely what we observed. We included caseload results (Table 2) only to show that they were sufficiently consistent with our primary findings to show predictive validity of our primary results (i.e., based on the number of operative workdays). A statistical consequence of the high variability among surgeons in cases per month (leading to wide confidence intervals) is that an absence of raw differences between genders based on case counts should not be interpreted as showing no bias (i.e., substantive risk of a Type II error). One may draw such a conclusion validly only if multivariable-adjusted confidence intervals are calculated, compensating for outliers, and if those confidence intervals are narrow. Unless an organization wants to make such efforts, we recommend that internal work assessing the potential presence of gender-based bias affecting surgical productivity rely on the endpoint of differences in the number of operative workdays, not differences in the number of cases.

Our novel endpoint of operative workdays per month is practical because it is easy to compare among surgeons, and, inherently, there cannot be large outliers because the number of operating workdays per month is limited. However, a drawback is that there are few earlier findings with our studied endpoint to make comparisons.

Novelty of study findings and comparisons with earlier studies

The general observation that female surgeons have “fewer” operative days and cases than male surgeons is not relevant to benchmarking and guiding individual multispecialty groups. What sets our work apart are the specific quantitative findings. With that caveat, our findings are consistent qualitatively with previous studies, summarized below [13-16].

Obstetricians-gynecologists in the United States completed socioeconomic surveys in 2003 [13]. Female surgeons worked 9% fewer total hours per week and performed 28% fewer procedures than their male counterparts [13]. Our study similarly found fewer workdays per month (Table 1, P < 0.0001) and cases per month (Table 2, P < 0.0001) for female surgeons.

**Figure 2:**
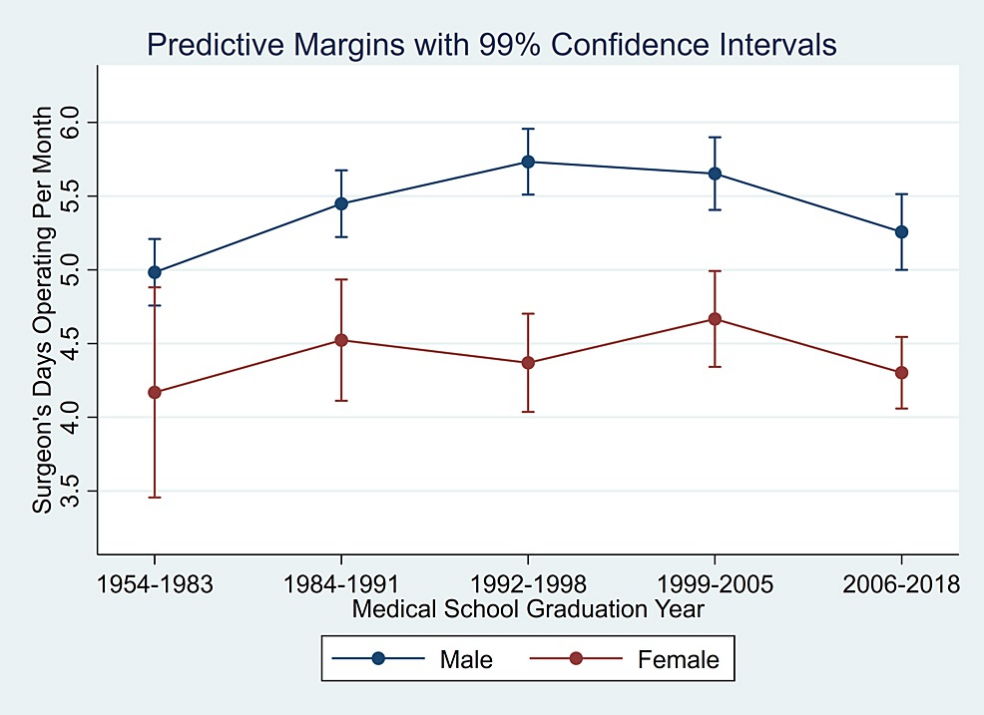
Predictive margins for workdays with at least one elective case among male and female surgeons calculated for each of the quintiles of the year of medical school graduation. Stata commands and output of the full regression model are in the Stata output at https://FDshort.com/SurgFMYr. The calculations used the observed population estimates for fractions of the population in each of the 16 different surgical specialties studied and among the 12 different quarters of data. Standard errors were estimated for coefficients using robust variance estimation with clustering among the 609 facilities. Then, the displayed 99% confidence intervals for the predicted mean (expected) values were calculated using the delta method. The important observation from the figure is that there are significant differences between genders for all years of graduation, and if more complicated models were applied that would be to quantify the significant interaction for a greater, not lesser, difference in the mid-career cohort (i.e., not relevant to study’s managerial application). The population was 8,472 surgeons, of whom 20.4% (1,724) were female. The percentages female among quintiles, from left to right, were 20.6% (1,742), 21.1% (1,789), 19.2% (1,625), 18.7% (1,585), and 20.4% (1,731). Treating the 8,472 surgeons as random effects to obtain an overall mean equally weighted for each surgeon, the result was 1.15 (standard error 0.01) operative workdays per week or 5.00 (standard error 0.03) workdays per month.

Obstetricians in Washington state were compared based on 1999 medical licensure data and counts of their delivered babies’ birth certificates [14]. Female obstetrician-gynecologists had an average of 4.1 hours per week fewer total professional hours than men, after adjusting for age [14]. The smallest ratios of female to male obstetricians for multiple endpoints (e.g., total professional hours, direct patient care hours, and the average number of deliveries per year) were among the 40- to 49-year age group compared with the 30- to 39-year or 50- to 59-year age groups [14]. This matches our result of a statistically significant but small interaction (see Figure 2 and the Stata output at https://FDshort.com/SurgFMYr) with male versus female differences being accentuated mid-career.

The 2017 billings of otolaryngologists in the United States were studied [15]. Female otolaryngologists billed fewer procedures than male otolaryngologists. This trend continued even after adjusting for year of medical school graduation and excluding female otolaryngologists of childbearing age; each comparison was reported as P < 0.001 [15]. The similarity of this and the other [13,14] study to our findings, qualitatively, show that our results of fewer operative workdays per month and fewer cases among female surgeons were expected. The novel value of our work is the quantification and confidence intervals that multispecialty groups can use to learn whether the level of deviation present in their practice is unusually large.

If childbearing years account for the small but significant differences in operative workdays between female and male surgeons, then female surgeons who are mid-career (i.e., older than median age when pregnant) would be expected to have smaller differences in workload compared to male surgeons than female surgeons of different ages. Our results are the opposite, with significant interaction for a greater, not lesser, difference based on gender in the mid-career cohort (see Figure 2 and the Stata output at https://FDshort.com/SurgFMYr). Our data supply no insight as to the mechanism (e.g., whether this dichotomy is caused by parenting responsibilities of older children including teenagers). However, our finding from Florida is consistent with findings from the United Kingdom in a study of the retirement of physicians who graduated in 1974 or 1977 [16]. Significantly more female physicians than male physicians, and specifically female surgeons, had retired by 2014 (i.e., 37 to 40 years since graduation) [16].

Earlier studies provide no basis for adding data on gender differences in research production (e.g., peer-reviewed publications) to counterbalance the observed differences in clinical production [17,18]. Twenty-five universities in the United States had faculty surveyed electronically [17]. Research productivity measured by the h‑index [19] was less among female than male faculty overall and for each of the four academic ranks of assistant professor, associate professor, professor, and chair [17]. In another study, surgical departments were audited at three large universities in the United States [18]. Counts of articles published were less among female than male faculty overall and for each rank of assistant professor, associate professor, and professor [18].

Additional considerations of multispecialty group surgeons’ productivity

Although we encourage multispecialty groups to compare operative workdays among surgeons based on gender to screen for systematic bias, another important issue from the perspective of perioperative managers is that surgeons in Florida collectively averaged relatively few (1.15, SE 0.01) operative workdays per week (Figure 2). There was a mean of 1.88 (SE 0.01) cases on each of those days, calculated after excluding ophthalmologists. These findings are unlikely artifactual or atypical. The observation of an average of two to three elective surgical cases per week among surgeons and proceduralists relying on anesthesia services has similarly been found in studies of three university hospitals [10,20,21], a large community hospital [22], a citywide health system [23], statewide in Iowa [24,25], and statewide in Florida [26,27], both among adult and pediatric surgeons [28].

The population of the United States aged 75 years and older is projected to increase by 74% between 2019 and 2034 [29]. Yet, the supply of surgeons is projected to decline unless retirements can be delayed (e.g., by two years) [29]. Conceptually, instead of retiring, some surgeons might be persuaded alternatively to work part-time [30]. However, our findings show that although the oldest cohort of surgeons worked significantly fewer workdays per month (and performed fewer cases) than middle-aged surgeons, the reductions were smaller (e.g., 0.64 [SE 0.10] workdays per month) than the contrast in gender (see Figure 2 and Stata output at https://FDshort.com/SurgFMYr). Furthermore, from a survey of physicians in the United Kingdom, the odds of surgeons working less than full time were much less (one-tenth) than that of general practitioners and without differences based on gender [31]. Thus, comparable or greater benefit to operative production would be accrued if female surgeons had as many operative workdays as male surgeons.

Increasing surgical productivity is also important in the long term for anesthesiology departments because anesthesia workload depends principally on surgeons [32]. Many surgical facilities nationwide have far fewer than eight hours of cases per anesthetizing location per workday [33-38], especially for non-operating room anesthetizing locations [37,38]. One implication of our results for anesthesiology departments in multispecialty groups is to consider supporting efforts to reorganize multispecialty groups functionally by organ system pathology, rather than by medical specialty. For example, large orthopedic departments often include primary care sports medicine physicians. Such collaboration frees up surgeons from taking care of patients who can be managed medically, giving them more days to perform surgery (i.e., more anesthetics). Our finding from the state of Florida that the median surgeon operates only a few days per month suggests that greater use of such combinations of surgical and medical providers might be fruitful to increase surgeons’ operative workdays.

Limitations

We could not assess causal mechanisms in part because of the lack of publicly available data that could be linked to understanding the practices and policies of individual facilities and multispecialty groups. Consequently, although we have quantified overall differences between female and male surgeons in operative workdays (Table 1) and cases (Table 2), the state of Florida administrative data does not include the fields to explain the results fully. For example, we do not know how our results would differ if female surgeons did not delay childbearing disproportionately to male surgeons, did not suffer disproportionate harassment at work, and were equally satisfied in their work life [39]. In other words, we have no data, one way or the other, as to whether the differences detected (Tables 1, 2) represent highly prevalent systematic bias. However, this limitation does not affect our overarching goal to help individual, large, multispecialty groups evaluate the potential presence of gender-based bias in their organizations. Future research could evaluate changes over time including during the coronavirus disease 2019 pandemic.

We studied data from one state, albeit a large one. On the other hand, an advantage over the earlier studies of oral maxillofacial surgeons and otolaryngologists using the Medicare Provider data [15,40,41] is that all patients of all ages and insurers were included. Although we are unaware of administrative laws or policies specific to Florida that could influence our results, we cannot discount regional, cultural, and geopolitical differences resulting in gender stereotype acceptance that support and form unconscious bias and influence behaviors when interacting with female surgeons.

Our results were from the United States and may differ among countries, probably dependent on baseline work hours [42]. Several hundred academic physicians in Sweden, the Netherlands, and Austria were compared, all working at universities with implemented strategies to assure equal treatment of all employees [42]. Among female physicians, the mean (standard deviation) “working hours as physicians” were 43 (18) in Austria, 41 (13) in Denmark, and 39 (11) in Sweden. In contrast, among male physicians, hours were 54 (19) in Austria, 46 (13) in Denmark, and 39 (12) in Sweden [42]. Consequently, female physicians worked significantly fewer hours than male physicians in Austria but not Sweden [42]. In a recent study from Liberia, surgical practitioners averaged even fewer operative workdays per week than typical for the United States [10,20-25,43]. Consequently, differences between genders may be smaller.

Finally, the National Provider Identifier database reports two genders: “female” and “male” [5,6]. These are the options for self-reporting currently mandatory [5]. Gender identity could not be assessed. However, if the prevalence of gender identity differing from sex were ≤0.7% among surgeons [44], there would be no effect on our conclusions.

## Conclusions

Adjusting for covariates including specialty, female surgeons in Florida operated an average of 1.0 fewer days per month than male surgeons, with a 99% confidence interval of 0.8 to 1.2 fewer days. The mean differences were 0.8 to 1.4 fewer days per month among each of the five quintiles of years of graduation from medical school. What this shows is that an average difference of 1.2 or fewer days per month is the status quo. Suppose that a multispecialty group’s female surgeons have anesthetic (operative) cases, on average, far fewer days per month (e.g., three less) than the group’s comparable male surgeons (e.g., same specialty). Then such a large average difference between male and female surgeons would call for investigation of what might reflect systematic bias. While such a difference may reflect good flexibility of the organization, it could also show a lack of responsiveness (e.g., fewer referrals of procedural patients to female surgeons or bias when apportioning allocated operating room time).
